# Eutectic-Driven Recrystallization of Coamorphous Bicalutamide + Niclosamide Systems: Contrasting Stability above and below *T*_g_

**DOI:** 10.1021/acs.molpharmaceut.5c01958

**Published:** 2026-06-09

**Authors:** Paulina Poloczek, Justyna Knapik-Kowalczuk, Joanna Klimontko, Xue Han, Kohsaku Kawakami, Marian Paluch

**Affiliations:** † Institute of Physics, 49568 University of Silesia in Katowice, 75 Pułku Piechoty 1A, Chorzów 41-500, Poland; ‡ Research Center for Macromolecules and Biomaterials, 52747 National Institute for Materials Science, 1-1 Namiki, Tsukuba, Ibaraki 305-0044, Japan; § Graduate School of Science and Technology, University of Tsukuba, 1-1-1 Tennodai, Tsukuba, Ibaraki 305-8577, Japan

**Keywords:** bicalutamide, niclosamide, eutectic composition, coamorphous systems, physical stability, molecular mobility

## Abstract

Coamorphization is a promising strategy to enhance the physical stability of poorly water-soluble drugs. However, the relationship between composition, molecular dynamics, and long-term stability remains insufficiently understood. Here, we investigate coamorphous systems composed of bicalutamide (BIC) and niclosamide (NIC), focusing on how the eutectic composition defined in the crystalline state translates into molecular dynamics and long-term physical stability of the corresponding amorphous systems above and below the glass transition temperature (*T*_g_). Thermal analysis revealed a eutectic point in the close vicinity of 25 wt % NIC, enabling safe melt-based amorphization of NIC without thermal degradation. Broadband dielectric spectroscopy showed that the addition of NIC does not significantly affect the molecular mobility of amorphous BIC, as reflected by comparable *T*_g_ values oscillating around 328 K, fragility parameters in the range of 85–92, as well as similar α-relaxation dynamics across all compositions. Despite these similarities, the physical stability of the coamorphous systems exhibits a pronounced composition dependence. Under accelerated conditions in the supercooled liquid state (*T* > *T*_g_), the coamorphous system corresponding to the eutectic concentration displays the highest resistance to recrystallization. In contrast, long-term X-ray diffraction studies performed under glassy-state conditions (*T* < *T*_g_) reveal a different stability ranking. These findings indicate that the stabilization observed for compositions corresponding to the eutectic point depends on the thermal regime and emphasize the importance of evaluating physical stability under both supercooled liquid and glassy state conditions.

## Graphical abstract

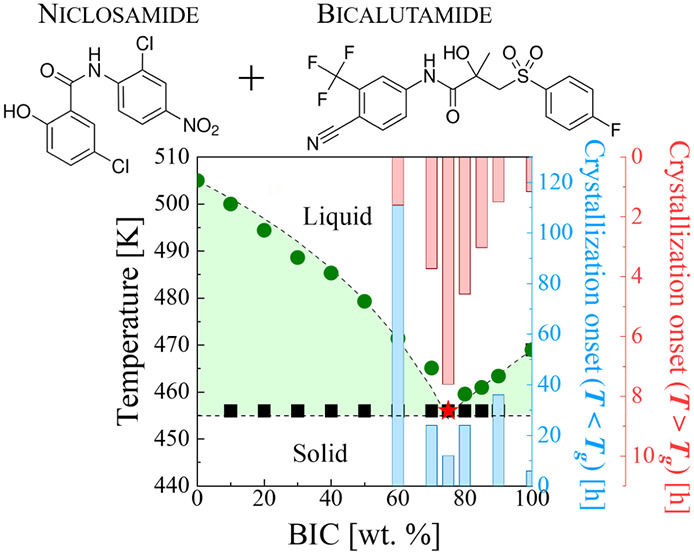

## 1 Introduction

Niclosamide (NIC) is an anthelmintic drug discovered in the 1950s, initially employed as a molluscicide.^1^ Approximately a decade later, its efficacy in treating tapeworm infections was established, leading to its approval by the United States Food and Drug Administration (FDA) for human use in 1982.^1,2^ In recent years, drug repurposing research has revealed NIC’s therapeutic potential in the management of conditions such as Parkinson’s disease, diabetes, and various viral and microbial infections.^1,2^ Of particular interest is its emerging role in oncology, where it has demonstrated anticancer activity, especially in the treatment of prostate cancer and other drug-resistant malignancies through combination therapy.^1–3^ A notable synergy has been observed when NIC is combined with bicalutamide (BIC), a nonsteroidal antiandrogen commonly employed in prostate cancer therapy due to its inhibition of androgen receptor (AR) activity.^3–7^ Recent studies have shown that this combination may help overcome resistance to both BIC and enzalutamide (ENZ), enhancing treatment outcomes and suppressing the progression of ENZ-resistant tumors.^3,5^

Despite their pharmacological potential, the clinical utility of this drug combination is severely limited by the poor aqueous solubility of both compounds, which adversely affects oral bioavailability.^4,8,9^ According to the Biopharmaceutics Classification System (BCS), both NIC and BIC are classified as class II drugs, characterized by low solubility and high permeability.^4,8–11^ This class encompasses many active pharmaceutical ingredients (APIs). To overcome this limitation, various strategies have been employed.^11,12^ One well-established approach is the conversion of crystalline compounds into their amorphous forms.^11–15^ Amorphous materials exhibit enhanced aqueous solubility due to the absence of long-range molecular order, which is associated with higher internal energy.^13–15^ However, the amorphous form of the BIC and NIC combination faces two significant limitations: thermal degradation during melt-based amorphization processes, resulting from the thermal instability of NIC (*T*_deg_ < *T*_m_) and insufficient physical stability, arising from the inherently high recrystallization tendency of amorphous BIC.^4,16^

During the manufacture of amorphous systems, particularly through techniques such as melt quenching or hot-melt extrusion, APIs are exposed to elevated temperatures that approach or exceed their melting points. Such thermal stress may induce degradation pathways, leading to a reduction in pharmacological efficacy and the formation of undesirable degradation products.^17–19^ Furthermore, the high free energy of amorphous phases renders them physically unstable, resulting in a propensity for recrystallization during processing or storage.^13,14,20–23^ This phenomenon is particularly evident in the case of BIC, as previous studies have demonstrated its tendency to recrystallize both from the supercooled liquid and the glassy state.^4,20^ Although multiple stabilization strategies have been investigated, coamorphization with low-molecular-weight excipients, such as the antiandrogen flutamide (FLU), remains one of the most promising approaches for maintaining the amorphous state of this API.^4,20,24–26^

In this paper, we present a comprehensive physicochemical characterization of the binary coamorphous systems composed of BIC and NIC. The study aims to elucidate how coamorphization affects the thermal behavior, molecular dynamics, and physical stability of the BIC + NIC system, both in the supercooled liquid and glassy states. Differential scanning calorimetry (DSC) and thermogravimetric analysis (TGA) were employed to investigate the thermal properties of both crystalline and coamorphous mixtures. The obtained results provided the basis for safe vitrification the BIC + NIC systems without thermal degradation. Broadband dielectric spectroscopy (BDS) was subsequently employed to investigate the temperature-dependent molecular mobility and to quantitatively determine the recrystallization kinetics of the studied systems under isothermal conditions performed at elevated temperature condition (*T* > *T*_g_). Finally, long-term stability tests under standard storage conditions (*T* < *T*_g_) were carried out using X-ray diffraction (XRD) to evaluate the preservation of the amorphous state over time. The combined results provide a molecular-level understanding of the interplay between composition, dynamics, and physical stability of coamorphous BIC + NIC system. The study revealed that the amorphous system with a composition corresponding to the eutectic point exhibits the highest physical stability when examined at temperatures above the glass transition (*T* > *T*_g_). Interestingly, such a trend was not observed when the systems were stored at the glassy state (*T* < *T*_g_).

## 2 Materials and Methods

### 2.1 Pure Compounds and Binary Mixtures

Bicalutamide (BIC, crystalline white powder, purity ≥ 99%, *M*_*w*_ = 430.37 g/mol) was purchased from Hangzhou Hyper Chemicals Limited, China. BIC is chemically described as *N*-[4-Cyano-3-(trifluoromethyl)­phenyl]-3-[(4-fluorophenyl)­sulfonyl]-2-hydroxy-2-methylpropanamide. Niclosamide (NIC, pale yellow crystalline powder, purity ≥ 99%, *M*_*w*_ = 327.12 g/mol) was purchased from Sigma-Aldrich, Germany. NIC is chemically describes as 2′,5-Dichloro-4′-nitrosalicylanilide. All materials were used as received. Binary mixtures were obtained by a mixing of neat substances using a mortar, and grinding them gently for 5 min at room temperature. The concentration of NIC in the compositions varied from 10 wt % to 90 wt % by weight. To achieve amorphous mixtures, the prepared powders were heated above their melting point and subsequently cooled to a glassy state.

### 2.2 Thermogravimetric Analysis (TGA)

Thermal stability of neat crystalline BIC and NIC was investigated by a Mettler TG 50 thermogravimetric analyzer (Mettler-Toledo, Switzerland) linked to a Mettler MT5 balance (Mettler-Toledo, Switzerland). The powder in open aluminum pans was placed in a furnace under nitrogen purge (50 mL/min) and heated at 10 K/min. Degradation of the sample was determined by the weight loss percentage.

### 2.3 Differential Scanning Calorimetry (DSC)

Thermal properties of neat, both crystalline and amorphous BIC and NIC, as well as their binary mixtures, were examined using Discovery DSC 250 system (TA Instruments Inc., USA) equipped with a refrigerated cooling system. For this purpose, powder samples of mass between 3 and 10 mg, either of a neat API or mechanical binary mixtures, were placed into aluminum crucibles (40 μL). As a reference, an empty aluminum crucible was utilized. All measurements were performed under a nitrogen purge of 50 mL/min. Instrument calibration for temperature and enthalpy was performed using indium and zinc standards. The melting point of neat substance and the solidus transition in the binary mixture was determined as the onset of the peak, whereas in the case of liquidus transition the peak maximum was detected. Glass transition temperature (*T*_g_) was identified as the midpoint of the heat capacity increment. All samples were measured with a heating rate of 10 K/min, while the cooling rate was 20 K/min. Each experiment was conducted at least in triplicate.

### 2.4 Broadband Dielectric Spectroscopy (BDS)

Molecular dynamics of neat amorphous BIC and its coamorphous mixtures containing 10, 25, and 40 wt % of NIC was investigated with a Novo-Control GMBH Alpha dielectric spectrometer (Novocontrol Technologies GmbH & Co. KG, Montabaur, Germany). Dielectric spectra were registered in a broad frequency range from 10^–1^ Hz to 10^6^ Hz. During the nonisothermal dielectric experiments the sample was heated (with a step of 2 K) from 331 K up to 367, 379, 389, and 379 K for neat BIC, BIC + 10 wt % NIC, BIC + 25 wt % NIC, and BIC + 40 wt % NIC, respectively. The temperature was controlled by a Quattro temperature controller with temperature stability better than 0.1 K. The examined systems were measured in a parallel-plate cell made of stainless steel (diameter of 15 mm, and a 0.1 mm gap provided by silica spacer fibers).

### 2.5 X-ray Diffraction (XRD)

XRD measurements of powdered samples, including neat crystalline BIC, neat crystalline NIC, neat amorphous BIC, as well as physical mixtures and coamorphous BIC + NIC systems containing 10, 20, 25, 30, and 40 wt % NIC, were performed using a Malvern Panalytical Empyrean diffractometer (Malvern Panalytical Ltd., Malvern, UK) equipped with a PIXcel^3D^ ultrafast solid-state hybrid detector and a Ni-filtered Cu Kα_1,2_ radiation source (λ = 1.5406 Å). Measurements were carried out at room temperature (oscillating around 293–296 K) in reflection mode using Bragg–Brentano geometry over a 2θ range of 5–45°. Prior to the first XRD measurement, amorphous samples were freshly prepared by melt quenching and analyzed immediately after vitrification. To ensure identical aging time, individual samples were prepared sequentially at intervals corresponding to the duration of a single XRD measurement. Following the initial measurement, the samples were stored in *SI SUBSTR 32 mm ZERO BACKGROUND SAMPLE HOLDERS* made from 32 mm diameter silicon single crystal substrates and placed in the built-in 15-position Sample Changer. Between subsequent XRD measurements, the samples were kept under continuously monitored temperature and relative humidity (35–38%) conditions.

## 3 Results and Discussion

### 3.1 Thermal Properties of Bicalutamide and Niclosamide Compositions

In the initial stage of this work, the thermal behavior of the neat (as received) BIC and NIC was examined by differential scanning calorimetry (DSC) and thermogravimetric analysis (TGA) to establish their fundamental thermal characteristics. The results—obtained during heating with a rate of 10 K/min—are presented in Figure 1. As shown in panel a of Figure 1, the DSC thermogram of neat BIC displays a single sharp endothermic event corresponding to melting at *T*_m_ = 465 K, which is in agreement with the literature data.^27^ When the melting temperature is compared with the temperature at which a 0.5% mass loss is observed on the TGA trace (considered a reliable indicator of the onset of thermal degradation), it becomes evident that BIC possesses a sufficient thermal window for safe amorphization by means of melt-based methods, such as vitrification. In contrast, panel b of Figure 1 presents the thermal profile of neat NIC. As can be noted the compound begins to melt at *T*_*m*_ = 503 K, what also agrees with the literature melting point of this API.^28,29^ It is worth pointing out that melting temperature overlaps with the NIC’s onset of thermal decomposition, clearly indicating that NIC is thermally unstable at its melting point, and attempts to obtain its amorphous form via melt-based methods may result in partially degraded or chemically impure material. Moreover, it should be emphasized that our experimental observations revealed that even when NIC melts (with evident partial degradation) it rapidly recrystallizes upon cooling. This crystallization behavior is documented in the Supporting Information (Figure S1), where DSC heating and cooling thermograms of neat NIC are presented. Therefore, the amorphization of NIC is highly challenging. On the one hand the compound degrades upon melting, and on the other hand it exhibits a strong tendency to recrystallize.

**Figure 1:**
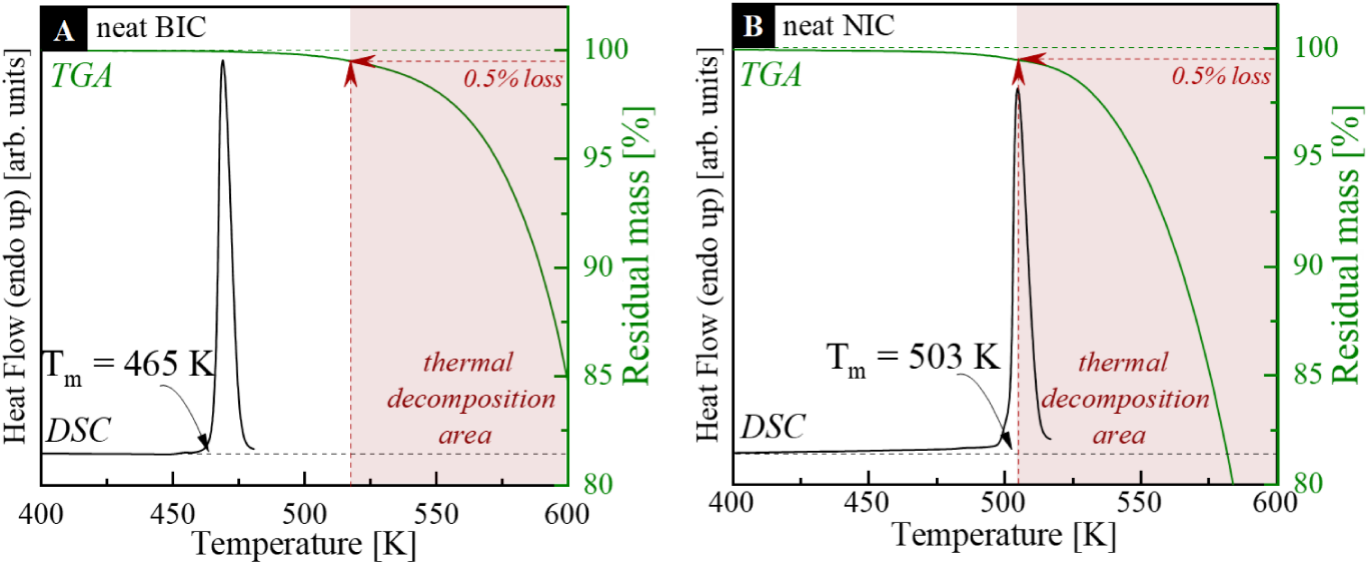
DSC (black) and TGA (green) heating scans of neat (A) BIC and (B) NIC recorded at a heating rate of 10 K/min. For BIC, a sharp melting endotherm at *T*_m_ = 465 K is observed well below the onset of thermal degradation, indicating a sufficient thermal window for melt-based amorphization. In contrast, for NIC the melting event at *T*_m_ = 503 K overlaps with the onset of thermal decomposition, as evidenced by a 0.5% mass loss (marked by dashed red lines), demonstrating its pronounced thermal instability. The shaded areas indicate the temperature ranges associated with thermal degradation. Additional data for representative binary system (BIC + 25 wt % NIC) are provided in the Supporting Information (Figure S2).

As it has been mentioned in the introduction, recent pharmacological studies have demonstrated a therapeutic synergy between BIC and NIC in the treatment of prostate cancer. Therefore, the next stage of our study focused on preparing and investigating the thermal properties of binary mixtures of these APIs, aiming to address the following questions: *How do BIC and NIC influence each other’s thermal behavior when combined? Can these compounds form a eutectic system that would reduce NIC’s melting temperature and consequently enable NIC’s amorphization in the presence of BIC via the melt-quenched method? If so, what is the eutectic concentration for the NIC and BIC system, and in which compositional ranges would amorphization be feasible (safe against thermal decomposition)?*

To answer these questions, binary mixtures of BIC and NIC at various weight concentrations were prepared (as described in the Materials section) and investigated using DSC. During the calorimetric experiments, the samples were heated from 298 to 520 K at a rate of 10 K/min. The obtained thermograms, alongside those of the neat APIs, are presented in Figure 2a.

**Figure 2:**
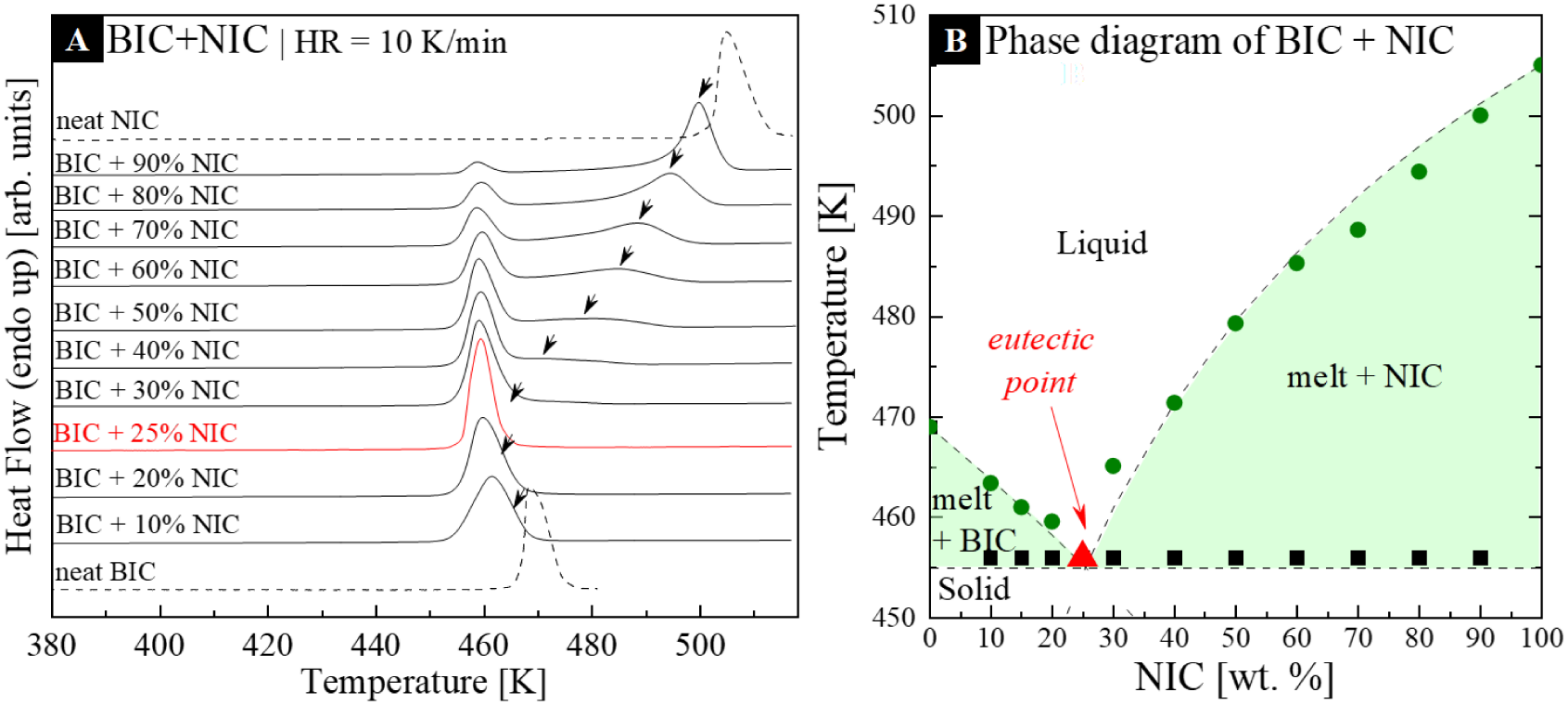
(A) DSC heating thermograms of crystalline BIC, NIC, and their binary mixtures recorded at a heating rate of 10 K/min. Depending on composition, the thermograms reveal either two distinct endothermic events corresponding to eutectic melting followed by melting of the excess component, or a single sharp endotherm characteristic of the eutectic composition (BIC + 25 wt % NIC, highlighted in red). (B) Phase diagram of the BIC + NIC system constructed from DSC data. Experimental melting points (symbols) are plotted together with liquidus lines calculated using the Schröder–van Laar equation (dashed lines).

As can be seen, the DSC traces of the binary BIC + NIC mixtures reveal distinct thermal behavior depending on the composition. Mixtures containing 90 to 30 wt % NIC exhibit two clearly separated endothermic events, corresponding to eutectic melting and the subsequent melting of the excess component.^30,31^ At 25 wt % NIC, a single, sharp and narrow endothermic peak is observed, characteristic of the eutectic composition, where both components melt simultaneously at a depressed temperature. Interestingly, at lower NIC concentrations (i.e., 20 and 10 wt %), only one broadened endothermic peak is observed. The broadening, relative to both the neat substances and the eutectic composition, suggests that this single thermal event is in fact an overlap of two unresolved transitions. To further verify this interpretation, additional DSC measurements were performed at a significantly reduced heating rate (0.5 K/min), which allowed improved separation of overlapping thermal events (see Figure S3 in the Supporting Information). To accurately characterize the thermal behavior of these compositions investigated with 10 K/min HR, peak deconvolution was performed using a dual-function fitting approach. The onset of the first endotherm and the maximum of the second endotherm were determined and subsequently used for the construction of the BIC + NIC phase diagram. Moreover, a complementary Tammann analysis was carried out to further support the determination of the eutectic composition (see Figure S4 in the Supporting Information). The resulting phase diagram is shown in Figure 2b, where the experimental data points are plotted together with the liquidus lines calculated using the Schröder–van Laar equation. This model describes the melting point depression in binary mixtures of crystalline solids based on the following formula:^32–34^

1 $$\ln\left(x_{i}\right)=\frac{\Delta H_{fus,i}}{R}\left(\frac{1}{T_{m,i}}-\frac{1}{T}\right)$$

where *x*_*i*_ is the mole fraction of component *i*, Δ*H*_*fus,i*_ and *T*_*m,i*_ represent the enthalpy of fusion as well as the melting temperature of the pure component, respectively, *T* is the temperature of the mixture at equilibrium, while *R* is the universal gas constant. It needs to be pointed out that the theoretical curves show good agreement with the experimental data confirming the eutectic point in the close vicinity of 25 wt % NIC and T = 455 K (based on the Tammann plot analysis (Figure S3), the eutectic composition was estimated to be approximately 26.4 wt % NIC). The observed eutectic behavior explains the visible melting point depression and proves that melt-based amorphization of NIC in the presence of BIC is feasible and thermally safe within the NIC concentration range of 0 to 40 wt %.

Considering both the melt-processing limitations of NIC-rich systems (i.e., thermal decomposition) and the described in the Introduction proven therapeutic benefits of BIC-dominated compositions, we focused our further investigations on amorphous binary BIC + NIC systems with a predominance of BIC. Selected mixtures containing 10, 15, 20, 25, 30, 35, and 40 wt % NIC were prepared by melting the crystalline powders in DSC crucibles followed by rapid cooling at a rate of 20 K/min. The resulting amorphous samples were subsequently reheated with a rate of 10 K/min. The recorded thermograms are presented in panel a of Figure 3, along with the trace of amorphous neat BIC for comparison.

**Figure 3:**
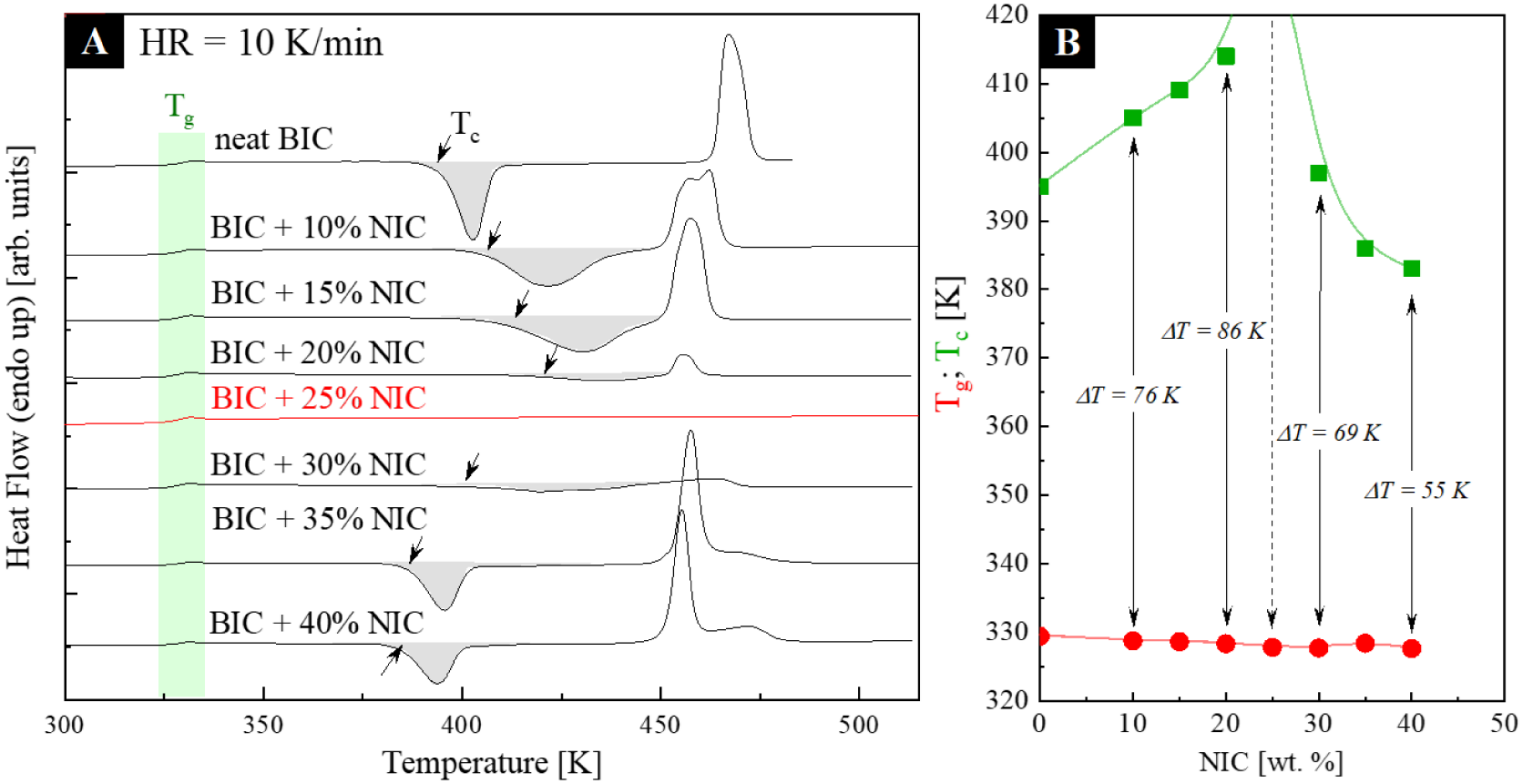
(A) DSC heating thermograms of neat amorphous BIC and coamorphous BIC + NIC systems recorded at a heating rate of 10 K/min. *T*_g_ of the samples is indicated by the green shaded region, while arrows mark the onset of cold crystallization (*T*_c_). Depending on composition, the systems exhibit pronounced differences in recrystallization tendency. Notably, the eutectic composition (BIC + 25 wt % NIC, highlighted in red) does not show any crystallization event within the experimental temperature window, indicating enhanced physical stability. (B) Composition dependence of the crystallization temperature (*T*_c_, green squares) and glass transition temperature (*T*_g_, red circles) for the investigated systems. The thermal gap Δ*T* = *T*_c_ – *T*_g_, indicated by arrows, serves as a measure of stability and reaches its maximum at the eutectic composition, confirming its superior resistance to recrystallization under nonisothermal conditions.

As can be seen, all binary mixtures exhibit a single glass transition event, with *T*_g_ values identical to that of neat BIC, i.e., *T*_g_ = 328 K. This value (*T*_g_ of neat BIC) agrees well with previously reported literature data.^4,20^ The observed invariance in BIC’s *T*_g_ in the binary compositions suggests that NIC and BIC possess similar *T*_g_ values. It is worth noting that this interpretation is further supported by the empirical two-thirds rule (*T*_g_ ≈ 2/3 *T*_m_), which allows for a rough estimation of the glass transition temperature of NIC. Using the reported melting temperature of NIC (*T*_m_ = 503 K), the estimated *T*_g_ is approximately 335 K. This value is in good agreement with the experimentally observed *T*_g_ range for the investigated systems, thereby reinforcing the conclusion that NIC and BIC possess similar glass transition temperatures. Upon further heating of neat BIC on the DSC thermogram, an exothermic peak was registered, confirming that the sample easily recrystallizes from the supercooled liquid state. This behavior is consistent with its classification as a second group of glass-formers (according to the classification system introduced in 2010 by Baird et al.), meaning that although it can be vitrified, it lacks the ability to maintain the amorphous state upon heating.^35^ Binary mixtures with 10 and 20 wt % NIC show slightly shifted to higher temperatures but still noticeable recrystallization exotherms, suggesting that NIC exerts a partial stabilizing effect. Interestingly, the composition being in the close vicinity to eutectic (25 wt % NIC) shows no recrystallization tendency throughout the entire heating scan. This stabilizing effect does not persist with increasing NIC content, and the DSC thermograms of systems containing 30 and 40 wt % of NIC again exhibit an endothermic event, reflecting the recrystallization tendency of these compositions. To better quantify the observed changes in recrystallization tendency, the thermal gap Δ*T* = *T*_c_ – *T*_g_ was calculated for each composition and is presented in panel b of Figure 3. This parameter serves as an indicator of kinetic stability of the investigated samples. The higher the Δ*T* value, the greater the resistance to recrystallization. As can be seen Δ*T* increases on both sides as the composition approaches the eutectic ratio (∼25 wt % NIC), for which no recrystallization was observed within the experimental window. This absence of crystallization suggests that the eutectic composition exhibits the highest physical stability among all studied amorphous systems. Such behavior, i.e., the maximal physical stability of the amorphous system coincident with the eutectic composition, is consistent with recent findings in the field of coamorphous pharmaceutical systems.^36,37^ To fully understand the origin of the observed improvement in the physical stability of BIC and NIC in their binary compositions, it is essential to go beyond thermal analysis. Thus, in the following sections, special attention is devoted to the investigation of molecular dynamics and relaxation processes in the studied systems, as well as to a detailed examination of how changes in composition influence the recrystallization behavior and overall physical stability of the coamorphous BIC + NIC mixtures at isothermal *T* conditions.

### 3.2 Investigation of the Impact of Coamorphization on the Molecular Dynamics of Neat BIC

To investigate the impact of amorphous NIC on the molecular dynamics of amorphous BIC, broadband dielectric spectroscopy (BDS) was employed. The analysis focused on the temperature dependence of the structural relaxation times and on the width of the structural relaxation peak. Notably, no well-defined secondary relaxation processes (such as Johari–Goldstein relaxation) were detected in the temperature range below *T*_g_ for any of the investigated systems. Instead, the dielectric response in the glassy state is dominated by a nearly constant loss (NCL) contribution. Therefore, the discussion is focused on the structural (α) relaxation, which constitutes the dominant dynamic process governing molecular mobility in these materials. The measurements were used also to verify the nonmonotonic changes in physical stability previously observed in the DSC experiments. For this purpose, neat amorphous BIC as well as BIC-based coamorphous systems containing 10, 25, and 40 wt % NIC were investigated. Importantly, the thermal stability issues of neat NIC described above are no longer a limiting factor for the investigated on BDS BIC + NIC compositions, as the combined systems exhibit sufficiently depressed melting temperatures, preventing thermal decomposition during melt-based processing. The measurements were carried out in the supercooled liquid and glassy state, heating the samples in 5 K step from 203 to 328 K and in 2 K steps from 331 K up to 367, 379, 389, and 379 K for neat BIC, BIC + 10 wt % NIC, BIC + 25 wt % NIC, and BIC + 40 wt % NIC, respectively, with the resulting dielectric loss spectra presented in Figure 4. As can be seen in all cases, a well-defined α-relaxation peak, associated with the cooperative structural dynamics, can be clearly identified. During heating, the α-relaxation shifts systematically toward higher frequencies, reflecting the acceleration of molecular dynamics with increasing temperature. In addition to the main α-process, a pronounced contribution from *dc*-conductivity is observed in the low-frequency region, which becomes more significant after the addition of NIC to the system. It is also worth noting that for each investigated sample, the intensity of the dielectric loss spectra at some point begins to decrease rapidly. This drop is directly associated with the recrystallization process, during which the number of dipoles contributing to the dielectric response is reduced (Δε ∼ *N*μ^2^, where *N* is the number of dynamically reorienting dipoles, while μ is their dipole moment).^38^ Thus, the observed reduction in Δε reflects a decrease in the fraction of molecules participating in structural relaxation due to their incorporation into the crystalline phase. As can be seen in Figure 4, the onset of the aforementioned intensity decrease, i.e., the beginning of sample recrystallization, strongly depends on the concentration of the BIC + NIC system. The earliest crystallization is observed for neat BIC, followed by the mixture containing 40 wt % NIC, then by the system with 10 wt % NIC, while the latest onset of recrystallization is recorded for the nearly eutectic composition (BIC + 25 wt % NIC). This nonmonotonic behavior is fully consistent with the trend observed and discussed in the previous section based on the calorimetric studies.

**Figure 4:**
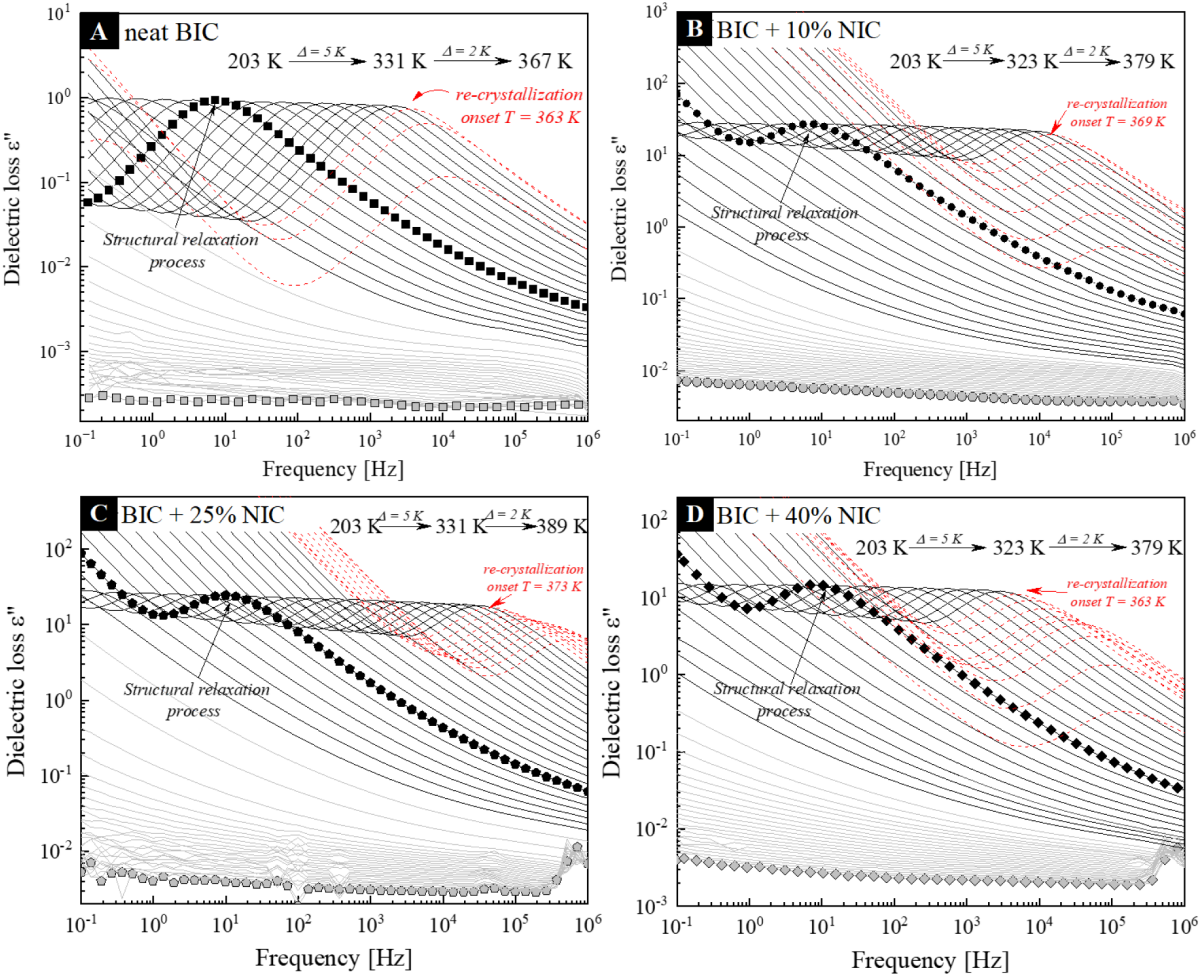
Dielectric loss spectra (ε″) recorded in the glassy state (*T* < *T*_g_—gray lines), and supercooled liquid region (*T* > *T*_g_—black lines) for (A) neat amorphous BIC and coamorphous BIC + NIC systems containing (B) 10 wt % NIC, (C) 25 wt % NIC, and (D) 40 wt % NIC. Measurements were performed upon heating in 5 K from 203 to 228 K and in 2 K temperature steps, starting from 331 K up to the temperatures indicated in each panel. Red dashed curves mark the spectra at which a rapid decrease in dielectric strength is observed, corresponding to the onset of recrystallization.

In order to assess whether, despite the absence of significant differences in *T*_g_, the investigated samples differ in the temperature dependence of the structural relaxation times (τ_α_(*T*)), the asymmetric α-relaxation peaks were fitted using the Havriliak–Negami (HN) function with conductivity correction, defined as:^39^

2 $$\varepsilon_{HN}^{*}\left(\omega \right)=\varepsilon^{\prime }\left(\omega \right)-i\varepsilon^{\prime \prime }\left(\omega \right)=\varepsilon_{\infty }+\frac{\Delta \varepsilon }{{[1+{\left(i\omega \tau_{HN}\right)}^{a}]}^{b}}+\frac{\sigma_{DC}}{\varepsilon_{0}i\omega }$$

The conductivity contribution was treated as an additive term and did not affect the reliability of the HN fits, as the α-relaxation peak remained well-defined and could be clearly separated from the low-frequency conductivity contribution. In eq 2, ε′(ω) and ε″(ω) are the dielectric responses in real and imaginary parts, respectively, ε_∞_ is the permittivity at high frequencies, Δε is dielectric strength, τ_*HN*_ represent the relaxation time, characteristic of the process, while α and β are peak shape parameters. Employing the obtained fitting parameters the structural relaxation time of the α-process (τ_α_) was calculated for each temperature in every investigated composition by using the following formula:^40–42^

3 $$\tau_{\alpha }=\tau_{HN}{[\sin\left(\frac{\pi \alpha }{2+2\beta }\right)]}^{-1/\alpha }{[\sin\left(\frac{\pi \alpha \beta }{2+2\beta }\right)]}^{1/\alpha }$$

It is worth pointing out that the values of τ_α_ calculated from the described above fitting procedure correspond to the inverse of the peak frequency (τ_α_ *=* 1/2π*f*_*max*_), where *f*_*max*_ denotes the frequency at which the maximum of the α-relaxation process occurs. The temperature dependences of the structural relaxation times τ_α_(*T*) determined for the investigated systems, i.e., neat amorphous BIC and the coamorphous BIC + 10, 25, and 40 wt % NIC mixtures, are compared in panel a of Figure 5. As can be seen, all systems exhibit nearly identical τ_α_(*T*) behavior, demonstrating that the addition of NIC to BIC does not practically modify its molecular dynamics, which most likely results from the very similar temperature dependence of the structural relaxation times of neat NIC to neat BIC. In order to parametrize this dependence, the Vogel–Fulcher–Tammann (VFT) equation was applied. The VFT relation is given by the following equation:^43–45^

4 $$\tau_{\alpha }\left(T\right)=\tau_{\infty }\exp\left(\frac{B}{T-T_{0}}\right)$$

where τ_∞_, *B*, and *T*_0_ are fitting parameters. Here, τ_∞_ represents the pre-exponential factor related to the vibrational time scale, *T*_0_ is the Vogel temperature corresponding to the divergence of relaxation time, and *B* = *DT*_0_, with *D* being the parameter that quantifies the deviation from simple Arrhenius behavior. The values of the obtained VFT parameters for both neat BIC and its coamorphous mixtures with NIC are summarized in Table 1. By extrapolating the VFT curves to τ_α_ = 100 s, the corresponding glass transition temperatures were determined for all investigated systems (*T*_g_ = 326 K). As can be seen, the *T*_g_ values derived from dielectric measurements are in good agreement with these obtained from calorimetric studies.

**Figure 5:**
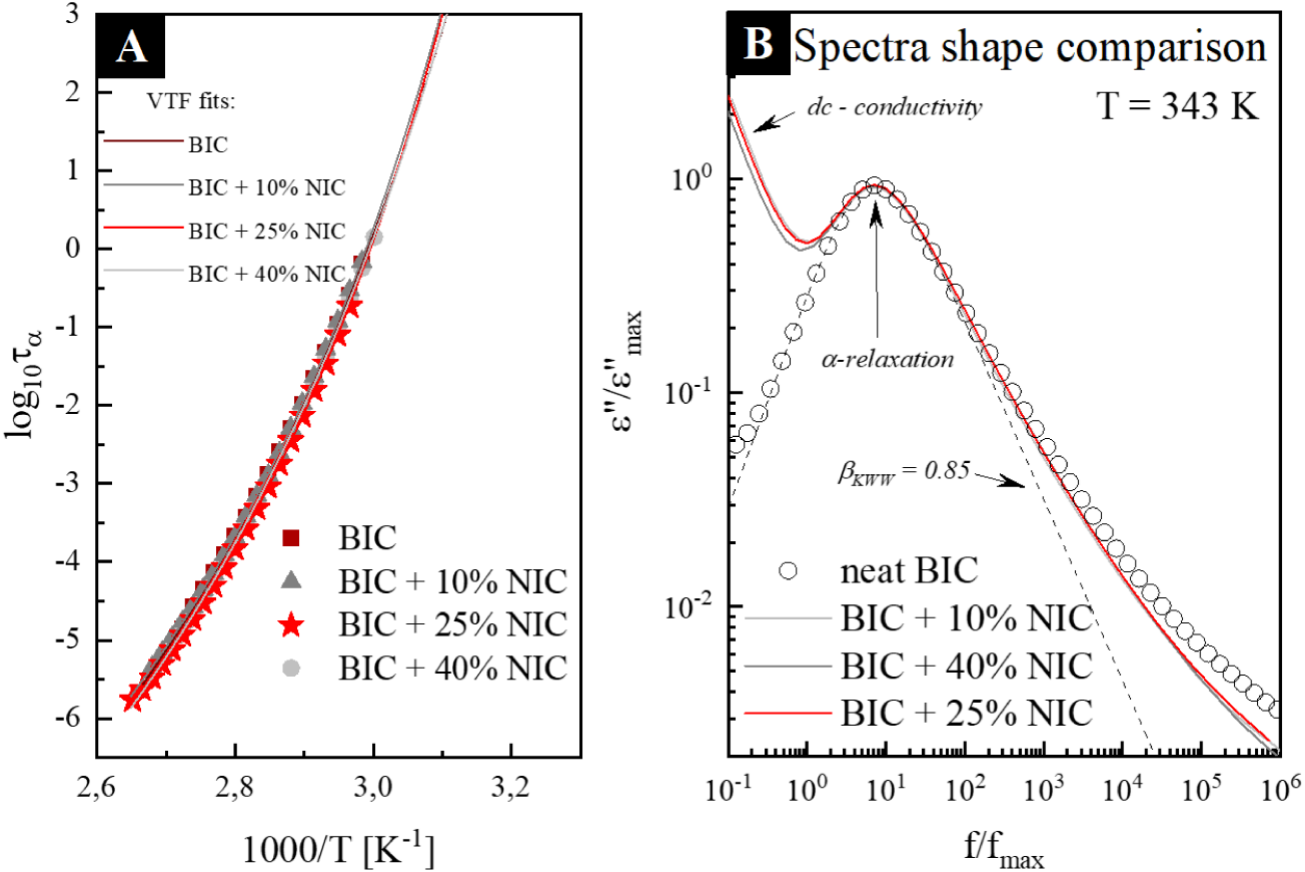
(A) Temperature dependence of the structural relaxation time (τ_α_) determined based on BDS measurements for neat amorphous BIC and coamorphous BIC + NIC systems containing 10, 25, and 40 wt % NIC. The solid lines represent fits to the VFT equation. (B) Comparison of normalized dielectric loss spectra (ε″/ε″_max_) recorded at *T* = 343 K for neat BIC and its coamorphous mixtures. All spectra exhibit a comparable α-relaxation peak shape, characterized by β_KWW_ = 0.85.

**Table 1:** VFT Fit Parameters Derived from Dielectric Data for Neat BIC and Coamorphous BIC + NIC Systems, along with the Corresponding *T*_g_ Values Determined from BDS, and *m*_p_

Sample:	log τ_∞_ [s]	*B* = *DT*_0_ [K]	*T*_0_ [K]	*T*_g,BDS_ [K]	*m*_p_
Neat BIC	–16.4 ± 0.2	3015 ± 98	254.4 ± 1.9	325.5	85
BIC + 10% NIC	–15.1 ± 0.2	2452 ± 67	264.0 ± 1.2	326.3	89
BIC + 25% NIC	–14.7 ± 0.2	2235 ± 82	265.6 ± 1.5	325.9	92
BIC + 40% NIC	–16.3 ± 0.3	2919 ± 141	256.3 ± 2.2	325.5	86

In the next part of the analysis of the dielectric loss spectra, the shape of the structural relaxation peaks was compared for all investigated systems. The spectra selected for this purpose, recorded at T = 343 K, are presented in panel b of Figure 5. As can be seen, the width of the dielectric loss peaks for all examined materials—neat BIC and its mixtures containing 10, 25, and 40 wt % NIC—can be satisfactorily parametrized using the same shape parameter β_KWW_ = 0.85 suggesting comparable degree of dynamic heterogeneity among the samples.^46^ The only noticeable difference between the compared spectra is the previously discussed increased contribution from *dc*-conductivity, which becomes more pronounced upon the addition of NIC.

A comparative analysis of the dielectric loss spectra for neat amorphous BIC and its coamorphous mixtures containing 10, 25, and 40 wt % NIC that has been presented in this section revealed only minor differences among the investigated samples. The temperature dependence of the structural relaxation time (τ_α_(*T*)), as well as the glass transition temperature (*T*_g_), the fragility parameter (*m*_p_), and the stretching parameter (β_KWW_) were found to be nearly identical for all systems.^12,13,41,47^ Considering that molecular dynamics often play a crucial role in governing the physical stability of amorphous pharmaceutical systems, the invariance of these parameters could suggest a comparable tendency toward recrystallization of all investigated systems. However, as demonstrated by the experimental data, the samples begin to recrystallize at different temperatures during heating, indicating that they differ significantly in terms of physical stability. This observation is fully consistent with the results obtained from calorimetric studies i.e., both DSC and BDS revealed a nonmonotonic dependence of recrystallization tendency on the NIC content in the binary amorphous BIC + NIC systems. It should be noted, however, that these conclusions are based solely on nonisothermal measurements. Therefore, to verify whether the same trend persists when the samples are maintained under identical thermal conditions, isothermal stability studies were carried out, as presented in the following sections of this work.

### 3.3 Physical Stability Studies of BIC + NIC Coamorphous Systems under accelerated *T* (*T* > *T*_g_) Conditions

To evaluate the physical stability of the amorphous BIC and its coamorphous mixtures containing 10, 15, 20, 25, 30, and 40 wt % of NIC at isothermal conditions, and in this section particularly at temperatures above the glass transition, time-resolved dielectric measurements were performed. Figure 6 presents the evolution of the dielectric loss spectra recorded at *T* = 348 K for two representative samples: neat BIC (panel a) and for the amorphous composition of BIC and NIC having eutectic concentration (i.e., BIC + 25 wt % NIC) (panel b).

**Figure 6:**
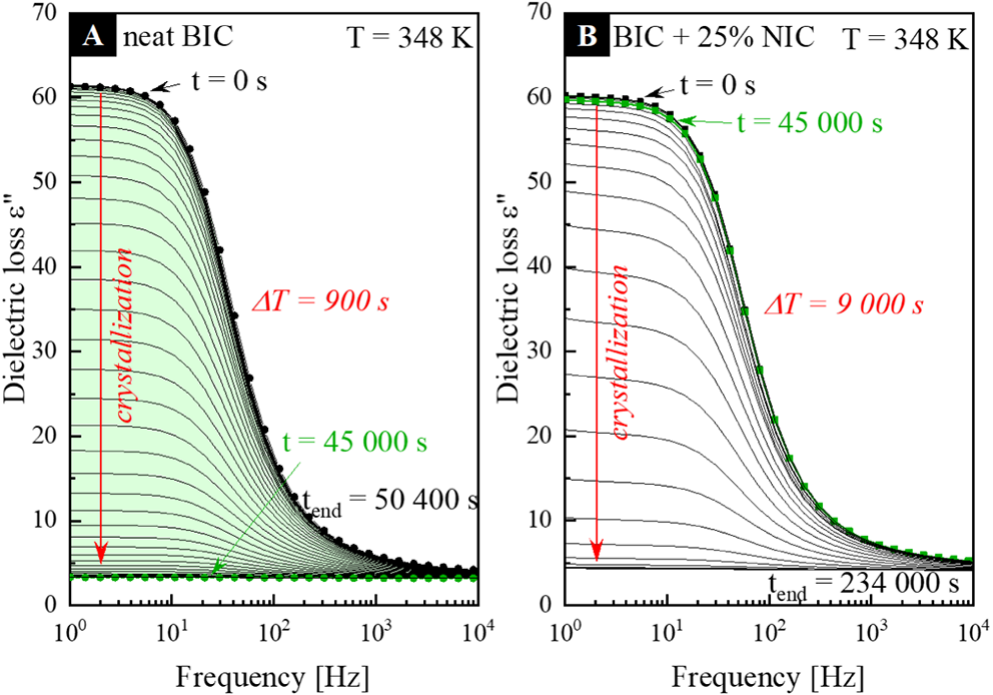
Time evolution of dielectric loss spectra (ε″) recorded at *T* = 348 K for (A) neat amorphous BIC and (B) the coamorphous eutectic composition (BIC + 25 wt % NIC). The measurements illustrate the recrystallization process monitored isothermally by BDS. *t*_end_ denotes the total experimental time, extending beyond the completion of recrystallization.

As can be seen, both systems show a gradual decrease in dielectric strength (Δε ∝ *N*·μ^2^) with increasing measurement time, which reflects the progressive loss of dipolar dynamics associated with recrystallization. In the case of neat BIC, the dielectric signal begins to decay rapidly after approximately 8 100 s, and the recrystallization process is completed within 45 000 s. In contrast, the amorphous mixture having the eutectic concentration remains stable for a significantly longer period i.e., the onset of recrystallization is strongly delayed. In panel b of Figure 6, the spectrum recorded at *t* = 45 000 s (i.e., the time at which neat BIC becomes fully recrystallized) is highlighted in green; at this *t* condition, in the case of the BIC + 25 wt % NIC mixture, the recrystallization process has only just begun, and the complete loss of dielectric response occurs after 213 000 s.

As was noted above, the results presented in Figure 6 are representative examples of the time-dependent dielectric response recorded for neat BIC and for the coamorphous BIC + NIC mixture corresponding to the eutectic concentration. However, analogous isothermal experiments were also performed for several other compositions of the BIC + NIC system in order to systematically evaluate the concentration-dependent physical stability. The dielectric data obtained for all studied mixtures were analyzed in terms of the normalized dielectric strength, ε’_*N*_(*t*) which represents the normalized progress of the recrystallization process, where ε’_*N*_(*t*) = 0 corresponds to the fully amorphous initial state and ε’_*N*_(*t*) = 1 corresponds to the fully recrystallized state. The normalization procedure was performed according to the relation:^48,49^

5 $${\varepsilon^{\prime }}_{N}\left(t\right)=\frac{\varepsilon^{\prime }\left(0\right)-\varepsilon^{\prime }\left(t\right)}{\varepsilon^{\prime }\left(0\right)-\varepsilon^{\prime }\left(\infty \right)}$$

where ε′(0) and ε′(∞) correspond to the initial and final permittivity values, respectively, and ε′(*t*) denotes the permittivity recorded at a given time. This normalization facilitates a direct comparison of crystallization kinetics across samples having different ε_*s*_ values. The resulting dependences are presented in Figure 7.

**Figure 7:**
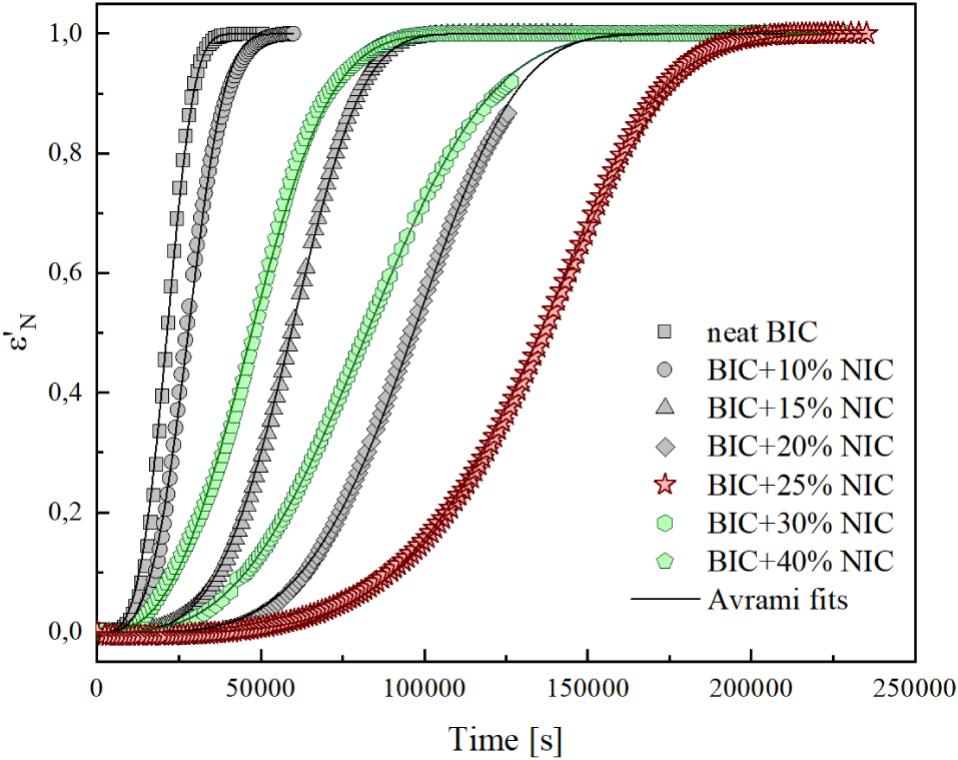
Normalized dielectric strength (ε′_N_) as a function of time recorded at *T* = 348 K for neat amorphous BIC and coamorphous BIC + NIC systems with different niclosamide contents. The decrease in ε′_N_ reflects the progress of recrystallization under isothermal conditions. Symbols represent experimental data, while solid lines correspond to fits using the Avrami model.

As can be seen, all investigated systems reveal a characteristic sigmoidal shape of the ε’_*N*_(*t*) curves, typical for crystallization processes governed by nucleation and growth mechanisms. In order to describe and compare the crystallization behavior of all the investigated samples, their kinetic curves were fitted using the Avrami model, which is one of the most established approaches for studying isothermal crystallization processes. The model is expressed by the following equation:^50^

6 $${\varepsilon^{\prime }}_{N}\left(t\right)=1-\exp\left(-kt^{n}\right)$$

where *k* denotes the crystallization rate constant and *n* represents the Avrami exponent, which reflects the dimensionality of crystal growth and the nature of the nucleation process. In most cases, the *n* parameter typically ranges between 2 and 3, however, in the present study, *n* values were found to vary within a broader range of 2 to 5, increasing as the composition approached the eutectic ratio. This trend suggests that the mechanism of crystallization becomes more complex near the eutectic composition, as additionally evidenced by deviations from ideal linear behavior observed in the linearized Avrami plots presented in Figure S5 (Supporting Information). Therefore, the Avrami analysis presented here should be treated as a semiquantitative description of the overall recrystallization behavior rather than a strict mechanistic interpretation based on an ideal single-step crystallization model. The corresponding values of the Avrami exponent and crystallization rate constant, are summarized in Table 2. It is worth pointing out that although the investigated systems exhibit very similar molecular dynamics, as evidenced by comparable glass transition temperatures, fragility parameters, and structural relaxation times, their crystallization kinetics differ significantly. This indicates that the crystallization process is not governed solely by molecular mobility. In general, crystallization kinetics are determined by two competing processes: nucleation and crystal growth. While structural relaxation reflects molecular mobility and is closely related to crystal growth, nucleation is strongly influenced by local structural organization and intermolecular interactions. In the present system, the addition of NIC does not significantly affect the α-relaxation dynamics of BIC, suggesting that molecular mobility remains largely unchanged. However, the pronounced differences in crystallization half-times indicate that nucleation processes are strongly composition-dependent. In particular, the eutectic composition exhibits the highest resistance to crystallization, which can be attributed to increased structural disorder and frustration, hindering the formation of stable crystalline nuclei. At higher NIC contents, the decrease in stability suggests that local structural arrangements may become more favorable for nucleation, despite similar molecular mobility.

**Table 2:** Avrami Crystallization Parameters (*k* and *n*) Obtained from Fits to the Isothermal Dielectric Crystallization Data Recorded at *T* = 348 K for Neat Amorphous BIC and Coamorphous BIC + NIC Systems with Different Niclosamide Contents Together with the Corresponding Crystallization Onset (*t*_onset_) and Half-Life (*t*_1/2_) Times

Sample	*k* [s^–n^]	*n*	*C* = *k* ^1/n^ [s^–1^]	*t*_onset_ [min]	*t*1/2 [h]
Neat BIC	4.94 × 10^–17^ ± 1.11 × 10^–18^	3.73 ± 0.02	3.6 × 10^–5^	70	5.71
BIC + 10% NIC	5.42 × 10^–17^ ± 9.54 × 10^–18^	3.63 ± 0.02	4.0 × 10^–5^	90	7.56
BIC + 15% NIC	1.32 × 10^–19^ ± 6.82 × 10^–21^	3.92 ± 0.00	1.5 × 10^–5^	182	16.41
BIC + 20% NIC	2.29 × 10^–22^ ± 1.32 × 10^–23^	4.31 ± 0.01	8.2 × 10^–6^	275	26.69
BIC + 25% NIC	7.78 × 10^–25^ ± 3.68 × 10^–26^	4.67 ± 0.00	4.4 × 10^–6^	456	38.01
BIC + 30% NIC	2.17 × 10^–16^ ± 1.12 × 10^–17^	3.15 ± 0.00	7.0 × 10^–6^	224	22.78
BIC + 40% NIC	3.41 × 10^–14^ ± 3.42 × 10^–15^	2.85 ± 0.01	1.7 × 10^–5^	97	13.32

Importantly, the observed trend in stability is identical to that found in the nonisothermal DSC and BDS experiments—the eutectic composition (BIC + 25 wt % NIC) exhibits the highest physical stability, while the physical stability of the remaining systems decreases with increasing deviation from the eutectic ratio. This nonmonotonic concentration dependence again confirms that the maximum stabilization effect arises when the intermolecular interactions and molecular packing reach their optimal balance, characteristic for the eutectic composition. To enable a quantitative comparison of the physical stability among all investigated materials, the crystallization onset (*t*_onset_) and half-life (*t*_1/2_) were determined and compared in the Table 2. For a clearer illustration and to facilitate direct comparison of the differences in physical stability, the obtained *t*_onset_ and *t*_1/2_ values were visualized in Figure 8 in the form of a red bar chart superimposed on the phase diagram of the BIC + NIC system.

**Figure 8:**
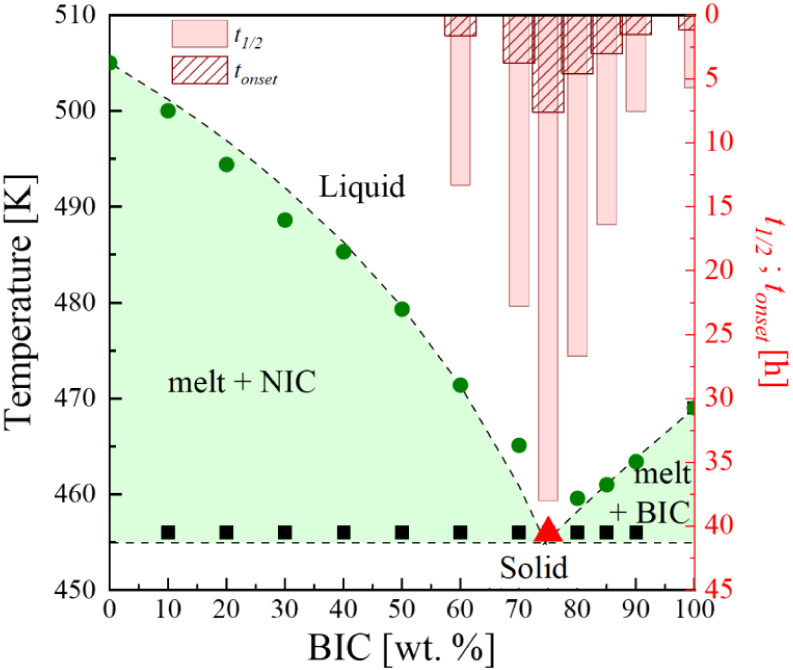
Red filled bars represent the crystallization half-times (*t*_1/2_), red patterned bars represent the crystallization onset (*t*_0_) determined from isothermal dielectric measurements at *T* = 348 K, illustrating the strong composition dependence of physical stability and the maximum stabilization at the eutectic composition. For clarity the bars are superimposed on the phase diagram of the BIC + NIC binary system constructed from DSC data.

The diagram clearly illustrates that the eutectic point, corresponding to 25 wt % NIC, coincides with the composition exhibiting the highest physical stability under accelerated conditions. It is worth noting that the observed correlation between the liquidus line of the crystalline BIC + NIC system and the crystallization half-life values of its amorphous counterparts suggests a strong relationship between the thermodynamic and kinetic properties of the system. The pronounced depression of the melting temperature at the eutectic composition facilitates molecular mixing and promotes the formation of a homogeneous amorphous phase, while the optimal intermolecular interactions between BIC and NIC appear to effectively suppress recrystallization. This combined thermodynamic-kinetic stabilization mechanism provides direct experimental evidence that the highest resistance to crystallization in coamorphous systems can be achieved when the composition corresponds to the eutectic point. It should be emphasized, however, that so far the physical stability of the investigated systems has been compared only in the supercooled liquid state.

### 3.4 Physical Stability Studies of BIC + NIC Coamorphous Systems under Standard Storage *T* (*T* < *T*_g_) Conditions

To verify whether the physical stability trend observed for the coamorphous BIC + NIC systems in the supercooled liquid state is preserved when the materials are stored in the glassy state—specifically, under standard storage conditions at *T*_room_—long-term stability tests were performed using X-ray diffraction (XRD). For this purpose, six representative samples were selected: neat BIC, BIC + 10 wt % NIC, BIC + 20 wt % NIC, BIC + 25 wt % NIC (corresponding to the eutectic composition), BIC + 30 wt % NIC and BIC + 40 wt % NIC. All these coamorphous systems were obtained by vitrification and subsequently powdered prior to the first XRD measurement, which was performed immediately after their preparation. The structural evolution of the materials was then monitored during storage: during the first 4 days, XRD measurements were carried out every 12 h, and afterward the measurement frequency was gradually reduced. The obtained diffraction patterns are presented in Figure 9.

**Figure 9:**
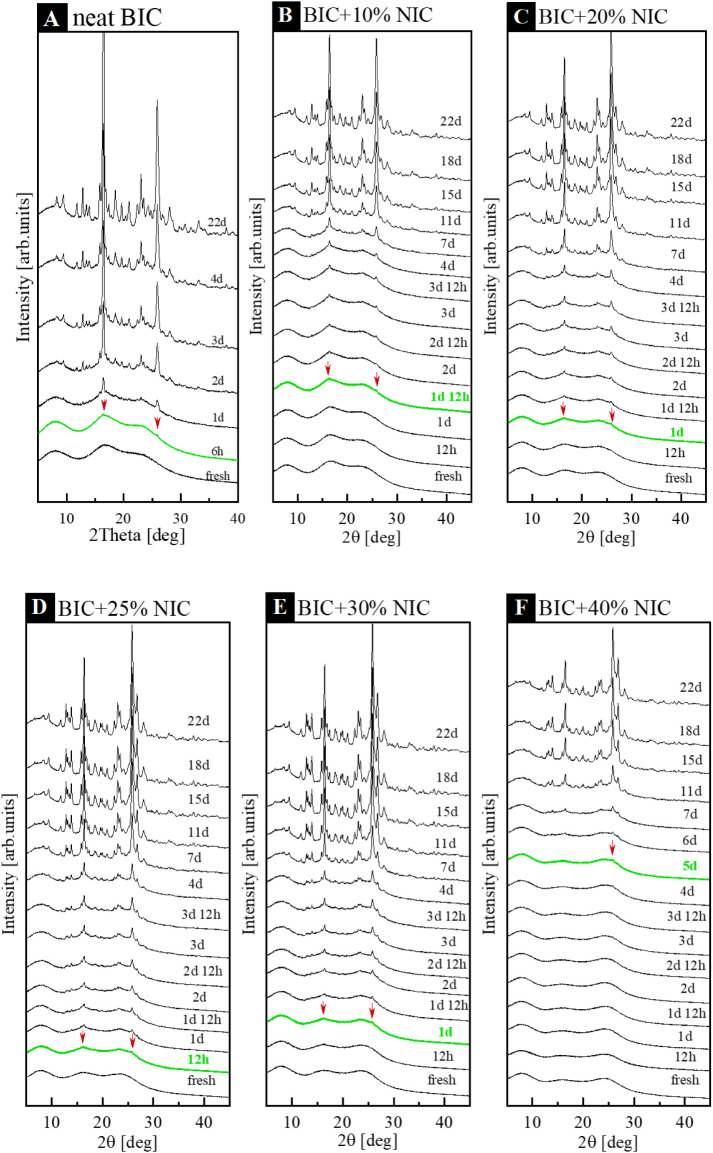
XRD patterns recorded during storage at *T*_room_ for (A) neat amorphous BIC and coamorphous BIC + NIC systems containing (B) 10 wt % NIC, (C) 20 wt % NIC, (D) 25 wt % NIC, (E) 30 wt % NIC, and (F) 40 wt % NIC. Diffraction patterns were collected immediately after sample preparation and at selected time intervals during storage. The appearance of sharp Bragg peaks indicates the onset of recrystallization. The green curves highlight the diffraction patterns recorded at the time when the first crystalline reflections were detected for each sample.

As expected, the least stable material among the investigated samples was neat amorphous BIC. The first weak but distinct sharp Bragg reflection was detected as early as 6 h after amorphization, indicating the onset of recrystallization. After 24 h, the XRD pattern of BIC already exhibited well-defined diffraction peaks, confirming advanced crystallization of the sample. The addition of 10 wt % NIC leads to a noticeable improvement in physical stability, with the first signs of recrystallization observed after approximately 36 h of storage. Interestingly, further increase in NIC content results in a nonmonotonic change in stability. For the sample containing 20 wt % NIC, the onset of recrystallization is accelerated, with the first small sharp Bragg peaks detected already after 24 h. Most notably, the composition corresponding to eutectic (i.e., BIC + 25 wt % NIC) exhibits the lowest physical stability in the glassy state, with the onset of recrystallization observed as early as 12 h after amorphization. Upon further increase in NIC content, the stability trend reverses again: the sample containing 30 wt % NIC begins to recrystallize after 24 h, whereas the system with 40 wt % NIC shows significantly improved stability, with the first detectable crystalline reflections appearing only after approximately 5 days of storage. This finding clearly indicates the absence of a direct correlation between the recrystallization tendency of the investigated coamorphous BIC + NIC systems when evaluated above and below the glass transition temperature i.e., under supercooled liquid (*T* > *T*_g_) and glassy state (*T* < *T*_g_) conditions. To the best of our knowledge, this is the first report showing that the composition-dependent physical stability of a coamorphous system corresponding to eutectic varies with temperature. These results highlight the importance of investigating physical stability under both accelerated and standard storage conditions, as the mechanisms governing recrystallization may differ substantially between the supercooled liquid and glassy states.

At the end of this section, it is worth discussing the nature of the solid material obtained after long-term recrystallization of the investigated systems. Figure 10 presents a comparison of the XRD patterns shown in Figure 9 for the BIC + NIC mixtures containing 10, 25, and 40 wt % NIC recorded after 1 year of storage, together with reference diffractograms of neat crystalline (as-received) BIC and NIC, as well as BIC recrystallized from the amorphous state after 22 days of storage. As can be seen, recrystallization in the investigated coamorphous systems involves both components. The observed diffraction features can be assigned to crystalline NIC in its original crystalline form and to BIC crystallizing into a form resembling that obtained upon recrystallization from the amorphous state, rather than the as-received crystalline BIC. This interpretation is supported by the presence of diffraction peaks characteristic of crystalline NIC and by reflections corresponding to the metastable, amorphous-derived polymorph of BIC. Importantly, no additional diffraction peaks appearing at new 2θ positions are detected for any of the investigated compositions. This observation indicates that recrystallization does not lead to the formation of a new crystalline phase, such as a cocrystal.

**Figure 10:**
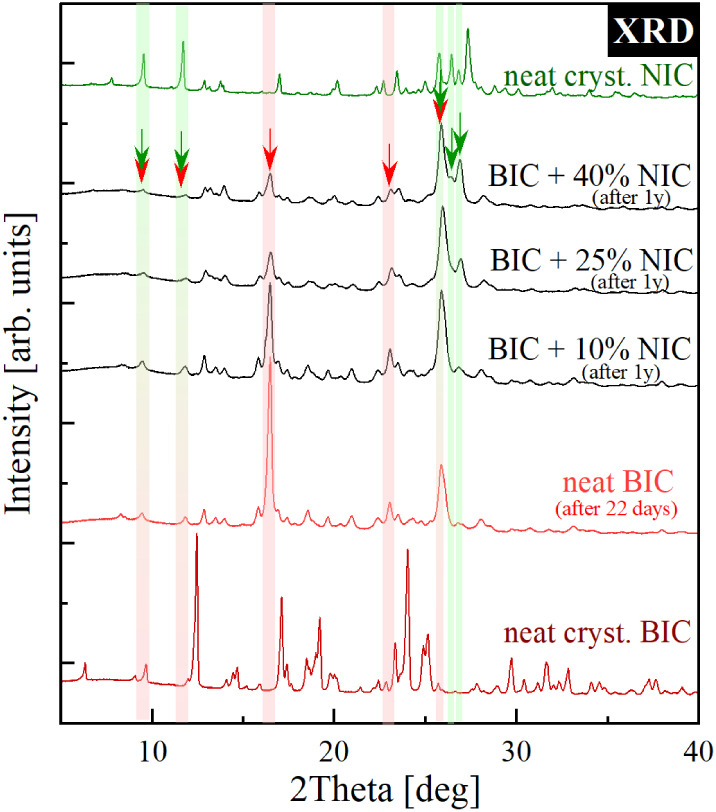
Comparison of XRD patterns recorded after 1 year of storage for coamorphous BIC + NIC systems containing 10, 25, and 40 wt % NIC with reference diffractograms of neat crystalline (as-received) BIC, neat crystalline NIC, and BIC recrystallized from the amorphous state after 22 days of storage. Shaded regions highlight characteristic diffraction features attributed to crystalline NIC (green) and to BIC recrystallized from the amorphous state (red).

To complement the XRD analysis and further assess the possible formation of new crystalline phases during long-term storage, additional DSC measurements were performed on samples stored for 29 days at room temperature. The corresponding thermograms, recorded upon heating (HR = 10 K/min), are presented in the Supporting Information (Figure S4) together with data for freshly prepared amorphous samples. All investigated systems exhibit a characteristic enthalpy overshoot at *T*_g_ due to physical aging, followed by cold crystallization and subsequent melting. Notably, the DSC traces reveal complex, multistep thermal behavior, including additional endothermic events at lower temperatures, which may suggest the in the glassy state formation of additional crystalline fractions (ex. cocrystal). However, due to the partially amorphous nature of the samples and the occurrence of recrystallization during heating (*T* > *T*_g_), these results cannot be unambiguously attributed to phases formed during isothermal storage at *T* < *T*_g_. Therefore, while DSC indicates the possibility of additional structural transformations, XRD remains the more reliable technique for phase identification under the investigated conditions. Importantly, the overall trends observed in DSC remain consistent with the XRD results, suggesting that recrystallization predominantly leads to the formation of the original crystalline components.

## 4 Conclusion

In this work, a comprehensive physicochemical study of the binary coamorphous systems composed of bicalutamide (BIC) and niclosamide (NIC) was carried out in order to investigate the relationship between composition, molecular dynamics, and physical stability. The constructed phase diagram of BIC + NIC confirmed the formation of a eutectic system with a eutectic point at 25 wt % NIC, enabling the vitrification of NIC in the presence of BIC without thermal degradation. The nonisothermal calorimetric studies revealed that the coamorphous composition having eutectic concentration exhibits the lowest tendency toward recrystallization among all investigated coamorphous binary systems. This result was further validated by nonisothermal dielectric investigations performed in the supercooled liquid state.

Broadband dielectric spectroscopy (BDS) showed that the addition of NIC to BIC does not significantly alter the molecular mobility of the system, as reflected by nearly identical values of *T*_g_, *m*_p_ as well as β_KWW_ across all compositions. Nevertheless, the physical stability of the amorphous systems strongly depends on composition, exhibiting a distinct nonmonotonic trend with a maximum at the eutectic ratio. Isothermal dielectric experiments demonstrated that the eutectic mixture possesses the longest crystallization half-life and the most delayed onset of recrystallization, confirming its superior stability under accelerated (*T* > *T*_g_) conditions. The strong correlation between the eutectic melting depression and the enhanced amorphous stability indicates that both thermodynamic and kinetic factors contribute cooperatively to the stabilization effect.

Interestingly, long-term X-ray diffraction (XRD) studies performed under standard storage conditions (*T* < *T*_g_) revealed a markedly different behavior, i.e., samples containing 20 and 30 wt % of NIC recrystallized after a comparable storage time, whereas the eutectic composition was no longer the most stable. To the best of our knowledge, this is the first report showing that the composition-dependent physical stability of a coamorphous system that forms a eutectic in its crystalline state varies with temperature. These findings demonstrate that the mechanisms governing recrystallization in the supercooled liquid and glassy states differs and underscore the necessity of assessing the physical stability of coamorphous pharmaceuticals under both accelerated and ambient storage conditions.

## Supplementary Material



## Notes

Additional thermal and structural characterization data supporting the conclusions of this study; including DSC heating/cooling thermograms of neat niclosamide; complementary DSC and TGA measurements of the BIC + NIC system; low-heating-rate DSC experiments enabling improved resolution of overlapping melting events; Tammann plot analysis confirming the eutectic composition; DSC thermograms of aged amorphous samples; and linearized Avrami plots used for semiquantitative analysis of crystallization kinetics (PDF)

## References

[1] Chen W., Mook R. A., Premont R. T., Wang J. (2018). Niclosamide: Beyond
an Antihelminthic Drug. Cell. Signalling.

[2] Kadri H., Lambourne O. A., Mehellou Y. (2018). Niclosamide, a Drug with Many (Re)­Purposes. ChemMedchem.

[3] Ren J., Wang B., Wu Q., Wang G. (2022). Combination of Niclosamide
and Current Therapies to Overcome Resistance for Cancer: New Frontiers
for an Old Drug. Biomed. Pharmacother..

[4] Pacult J., Rams-Baron M., Chmiel K., Jurkiewicz K., Antosik A., Szafraniec J., Kurek M., Jachowicz R., Paluch M. (2019). How Can We Improve the Physical Stability of Co-Amorphous
System Containing Flutamide and Bicalutamide? The Case of Ternary
Amorphous Solid Dispersions. Eur. J. Pharm.
Sci..

[5] Liu C., Armstrong C. M., Lou W., Lombard A. P., Cucchiara V., Gu X., Yang J. C., Nadiminty N., Pan C. X., Evans C. P., Gao A. C. (2017). Niclosamide
and Bicalutamide Combination Treatment
Overcomes Enzalutamide- and Bicalutamide-Resistant Prostate Cancer. Mol. Cancer Ther..

[6] Fradet Y. (2004). Bicalutamide
(Casodex®) in the Treatment of Prostate Cancer. Expert Rev. Anticancer Ther..

[7] Bohl C. E., Gao W., Miller D. D., Bell C. E., Dalton J. T., Kuriyan J. (2005). Structural
Basis for Antagonism and Resistance of Bicalutamide in Prostate Cancer. Proc. Natl. Acad. Sci. U. S. A.

[8] Vega D. R., Polla G., Martinez A., Mendioroz E., Reinoso M. (2007). Conformational Polymorphism in Bicalutamide. Int. J. Pharm..

[9] Lodagekar A., Borkar R. M., Thatikonda S., Chavan R. B., Naidu V. G. M., Shastri N. R., Srinivas R., Chella N. (2019). Formulation and Evaluation
of Cyclodextrin Complexes for Improved Anticancer Activity of Repurposed
Drug: Niclosamide. Carbohydr. Polym..

[10] () Kanfer, I. Report On The International Workshop On The Biopharmaceutics Classification System (BCS): Scientific And Regulatory Aspects In Practice; 2002. www.ualberta.ca/~cspswww.ualberta.ca/~csps. 12061340

[11] Rodriguez-Aller M., Guillarme D., Veuthey J. L., Gurny R. (2015). Strategies for Formulating
and Delivering Poorly Water-Soluble Drugs. J.
Drug Delivery Sci. Technol..

[12] Knapik J., Wojnarowska Z., Grzybowska K., Tajber L., Mesallati H., Paluch K. J., Paluch M. (2016). Molecular
Dynamics and Physical Stability
of Amorphous Nimesulide Drug and Its Binary Drug-Polymer Systems. Mol. Pharm..

[13] Grzybowska K., Paluch M., Grzybowski A., Wojnarowska Z., Hawelek L., Kolodziejczyk K., Ngai K. L. (2010). Molecular Dynamics
and Physical Stability of Amorphous Anti-Inflammatory Drug: Celecoxib. J. Phys. Chem. B.

[14] Grzybowska K., Chmiel K., Knapik-Kowalczuk J., Grzybowski A., Jurkiewicz K., Paluch M. (2017). Molecular Factors Governing
the Liquid
and Glassy States Recrystallization of Celecoxib in Binary Mixtures
with Excipients of Different Molecular Weights. Mol. Pharmaceutics.

[15] Knapik-Kowalczuk J., Gündüz M. G., Chmiel K., Jurkiewicz K., Kurek M., Tajber L., Jachowicz R., Paluch M. (2020). Molecular Dynamics, Viscoelastic
Properties and Physical
Stability Studies of a New Amorphous Dihydropyridine Derivative with
T-Type Calcium Channel Blocking Activity. Eur.
J. Pharm. Sci..

[16] Yang W., De Villiers M. M. (2005). Effect of 4-Sulphonato-Calix­[n]­Arenes and Cyclodextrins
on the Solubilization of Niclosamide, a Poorly Water Soluble Anthelmintic. AAPS J..

[17] Vasconcelos T., Marques S., Das Neves J., Sarmento B. (2016). Amorphous Solid Dispersions:
Rational Selection of a Manufacturing Process. Adv. Drug Delivery Rev..

[18] Zhao Y., Xie X., Zhao Y., Gao Y., Cai C., Zhang Q., Ding Z., Fan Z., Zhang H., Liu M., Han J. (2019). Effect of Plasticizers on Manufacturing Ritonavir/Copovidone
Solid
Dispersions via Hot-Melt Extrusion: Preformulation, Physicochemical
Characterization, and Pharmacokinetics in Rats. Eur. J. Pharm. Sci..

[19] Tran P., Pyo Y. C., Kim D. H., Lee S. E., Kim J. K., Park J. S. (2019). Overview of the Manufacturing Methods
of Solid Dispersion
Technology for Improving the Solubility of Poorly Water-Soluble Drugs
and Application to Anticancer Drugs. Pharmaceutics.

[20] Szczurek J., Rams-Baron M., Knapik-Kowalczuk J., Antosik A., Szafraniec J., Jamróz W., Dulski M., Jachowicz R., Paluch M. (2017). Molecular Dynamics, Recrystallization Behavior, and
Water Solubility of the Amorphous Anticancer Agent Bicalutamide and
Its Polyvinylpyrrolidone Mixtures. Mol. Pharm..

[21] Van
Den Mooter G. (2012). The Use of Amorphous Solid Dispersions: A Formulation
Strategy to Overcome Poor Solubility and Dissolution Rate. Drug Discovery Today: Technol..

[22] Jermain S. V., Brough C., Williams R. O. (2018). Amorphous Solid
Dispersions and Nanocrystal
Technologies for Poorly Water-Soluble Drug Delivery – An Update. Int. J. Pharm..

[23] Chmiel K., Knapik-Kowalczuk J., Jachowicz R., Paluch M. (2019). Broadband Dielectric
Spectroscopy as an Experimental Alternative to Calorimetric Determination
of the Solubility of Drugs into Polymer Matrix: Case of Flutamide
and Various Polymeric Matrixes. Eur. J. Pharm.
Biopharm..

[24] Abu-Diak O. A., Jones D. S., Andrews G. P. (2012). Understanding the Performance of
Melt-Extruded Poly­(Ethylene Oxide)–Bicalutamide Solid Dispersions:
Characterisation of Microstructural Properties Using Thermal, Spectroscopic
and Drug Release Methods. J. Pharm. Sci..

[25] Szafraniec-Szczęsny J., Antosik-Rogóż A., Knapik-Kowalczuk J., Kurek M., Szefer E., Gawlak K., Chmiel K., Peralta S., Niwiński K., Pielichowski K., Paluch M., Jachowicz R. (2020). Compression-Induced Phase Transitions
of Bicalutamide. Pharmaceutics.

[26] Antosik-Rogóż A., Szafraniec-Szczęsny J., Knapik-Kowalczuk J., Kurek M., Gawlak K., Paluch M., Jachowicz R. (2022). How Does Long-Term
Storage Influence the Physical Stability and Dissolution of Bicalutamide
from Solid Dispersions and Minitablets?. Processes.

[27] Gana I., Céolin R., Rietveld I. B. (2013). Bicalutamide Polymorphs i and II:
A Monotropic Phase Relationship under Ordinary Conditions Turning
Enantiotropic at High Pressure. J. Therm. Anal.
Calorim..

[28] Van
Tonder E. C., Maleka T. S. P., Liebenberg W., Song M., Wurster D. E., De Villiers M. M. (2004). Preparation
and Physicochemical Properties of Niclosamide Anhydrate and Two Monohydrates. Int. J. Pharm..

[29] Sanphui P., Kumar S. S., Nangia A. (2012). Pharmaceutical
Cocrystals of Niclosamide. Cryst. Growth Des..

[30] Rycerz L. (2013). Practical
Remarks Concerning Phase Diagrams Determination on the Basis of Differential
Scanning Calorimetry Measurements. J. Therm.
Anal. Calorim..

[31] Agarwal P., Svirskis D., Nieuwoudt M. K. (2024). Thermodynamic
and Spectroscopic Evaluation
of the Eutectic Mixture of Myristic Acid and the Local Anaesthetics,
Bupivacaine and Ropivacaine. RSC Pharm..

[32] Stott P. (1998). Transdermal
Delivery from Eutectic Systems: Enhanced Permeation of a Model Drug,
Ibuprofen. J. Controlled Release.

[33] Le
Minh T., Von Langermann J., Lorenz H., Seidel-Morgenstern A. (2010). Enantiomeric
3-Chloromandelic Acid System: Binary Melting Point Phase Diagram,
Ternary Solubility Phase Diagrams and Polymorphism. J. Pharm. Sci..

[34] Pyykkö P. (2019). Simple Estimates
for Eutectic Behavior. ChemPhyschem.

[35] Baird J. A., Van Eerdenbrugh B., Taylor L. S. (2010). A Classification System to Assess
the Crystallization Tendency of Organic Molecules from Undercooled
Melts. J. Pharm. Sci..

[36] Kissi E. O., Khorami K., Rades T. (2019). Determination
of Stable Co-Amorphous
Drug–Drug Ratios from the Eutectic Behavior of Crystalline
Physical Mixtures. Pharmaceutics.

[37] Knapik-Kowalczuk J., Kramarczyk D., Jurkiewicz K., Chmiel K., Paluch M. (2021). Ternary Eutectic
Ezetimibe-Simvastatin-Fenofibrate System and the Physical Stability
of Its Amorphous Form. Mol. Pharm..

[38] Strojewski D., Lalik S., Danède F., Górska N., Deptuch A., Marzec M., Willart J. F., Krupa A. (2024). Bosentan Monohydrate
and Sildenafil Base as Two Companions in Enabling Formulations. Int. J. Pharm..

[39] Havriliak S., Negami S. (1967). A Complex Plane Representation
of Dielectric and Mechanical
Relaxation Processes in Some Polymers. Polymer.

[40] Schönhals, A. ; Kremer, F. Analysis of Dielectric Spectra. In Broadband Dielectric Spectroscopy; Springer: Berlin Heidelberg, 2003, pp. 59–98. 10.1007/978-3-642-56120-7_3.

[41] Adrjanowicz K., Kaminski K., Paluch M., Wlodarczyk P., Grzybowska K., Wojnarowska Z., Hawelek L., Sawicki W., Lepek P., Lunio R. (2010). Dielectric Relaxation Studies and
Dissolution Behavior of Amorphous Verapamil Hydrochloride. J. Pharm. Sci..

[42] Rodrigues A. C., Viciosa M. T., Danède F., Affouard F., Correia N. T. (2014). Molecular
Mobility of Amorphous S-Flurbiprofen: A Dielectric Relaxation Spectroscopy
Approach. Mol. Pharm..

[43] Vogel H. (1921). Das Temperaturabhangigkeitgesetz
Der Viskosität von Flüssigkeiten. J. Phys. Z.

[44] Fulcher G. S. (1925). Analysis
of Recent Measurements of the Viscosity of Glasses. J. Am. Ceram. Soc..

[45] Tammann G., Hesse W. (1926). Die Abhängigkeit
Der Viscosität von Der Temperatur
Bie Unterkühlten Flüssigkeiten. Z. Anorg. Allg. Chem..

[46] Shamblin S. L., Hancock B. C., Dupuis Y., Pikal M. J. (2000). Interpretation of
Relaxation Time Constants for Amorphous Pharmaceutical Systems. J. Pharm. Sci..

[47] Kothari K., Ragoonanan V., Suryanarayanan R. (2015). The Role of Drug-Polymer Hydrogen
Bonding Interactions on the Molecular Mobility and Physical Stability
of Nifedipine Solid Dispersions. Mol. Pharm..

[48] Grzybowska K., Capaccioli S., Paluch M. (2016). Recent Developments in the Experimental
Investigations of Relaxations in Pharmaceuticals by Dielectric Techniques
at Ambient and Elevated Pressure. Adv. Drug
Delivery Rev..

[49] Knapik-Kowalczuk J., Tu W., Chmiel K., Rams-Baron M., Paluch M. (2018). Co-Stabilization of
Amorphous Pharmaceuticals—The Case of Nifedipine and Nimodipine. Mol. Pharm..

[50] Avrami M. (1939). Kinetics of
Phase Change. I General Theory. J. Chem. Phys..

