# Supplementary material for: Eutectic-Driven Recrystallization of Coamorphous Bicalutamide + Niclosamide Systems: Contrasting Stability above and below T g
Source: Mol Pharm. 2026 Jun 9;23(7):3641–53. doi: 10.1021/acs.molpharmaceut.5c01958 (PMC13343511; doi:10.1021/acs.molpharmaceut.5c01958)
Supplement: Supplementary file 1 [file mp5c01958_si_001.pdf]

Eutectic-driven recrystallization of co-amorphous bicalutamide + niclosamide systems: contrasting stability above and below T_g

Paulina Poloczek[1], Justyna Knapik-Kowalczuk[1,2]*, Joanna Klimontko[1], Xue Han[2,3], Kohsaku Kawakami[2,3], Marian Paluch[1]

[1]Institute of Physics, University of Silesia in Katowice, 75 Pułku Piechoty 1A, 41-500, Chorzów, Poland

[2]Research Center for Macromolecules and Biomaterials, National Institute for Materials Science, 1-1 Namiki, Tsukuba 305-0044, Ibaraki, Japan

[3]Graduate School of Science and Technology, University of Tsukuba, 1-1-1 Tennodai, Tsukuba 305-8577, Ibaraki, Japan

*e-mail: justyna.knapik-kowalczuk@us.edu.pl

Supporting information

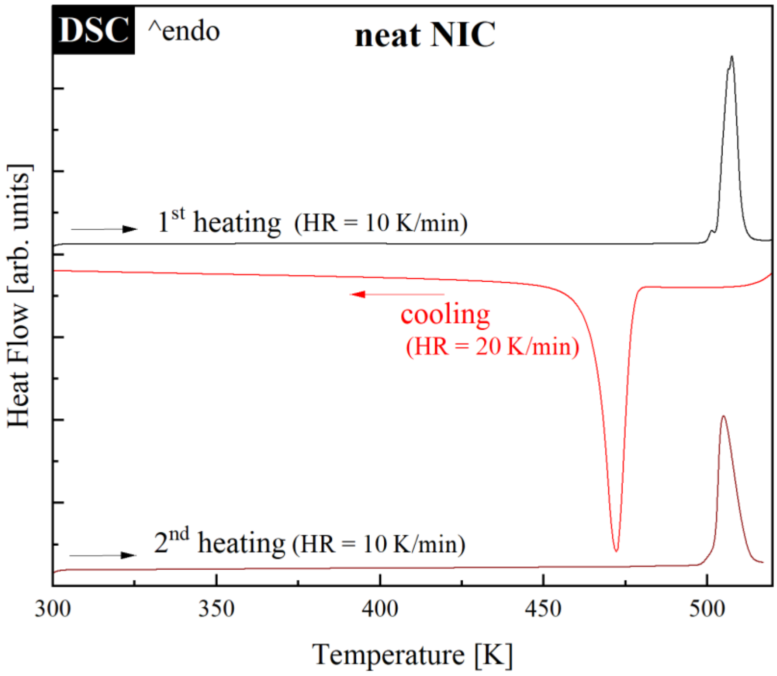

Figure S1. Differential scanning calorimetry (DSC) thermograms of neat niclosamide (NIC) recorded during the first heating (10 K·min⁻¹), subsequent cooling (20 K/min), and second heating (10 K/min). The first heating scan reveals a clear melting endotherm of crystalline NIC. Upon cooling from the melt, a pronounced exothermic peak corresponding to recrystallization is observed, indicating rapid crystallization of NIC. During the second heating, only the melting endotherm is detected, demonstrating that NIC does not remain in the amorphous state upon cooling at the applied rate and cannot be supercooled under the investigated conditions.

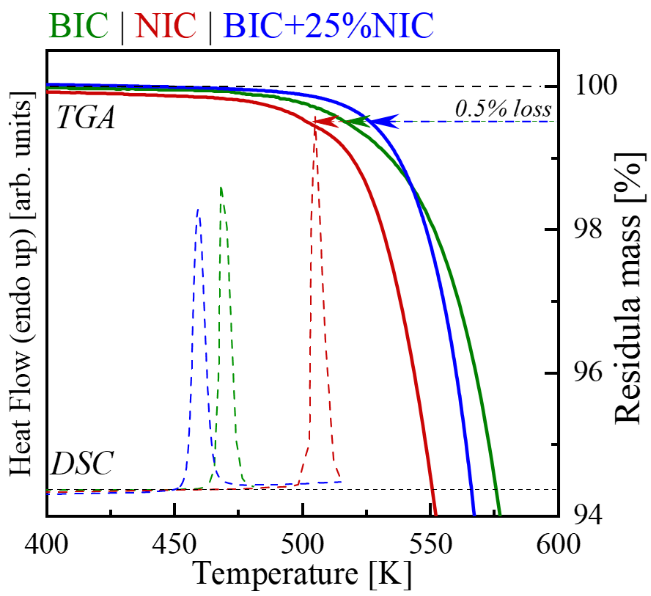

Figure S2. DSC (dashed) and TGA (solid) curves of neat BIC (green), neat NIC (red), and the representative binary system - BIC + 25 wt.% NIC (blue) recorded at a heating rate of 10 K/min. The comparison shows that the temperature corresponding to a given mass loss (e.g., 0.5%) is slightly shifted toward higher values for the binary system relative to the pure components, suggesting slight improved thermal stability of the mixture.

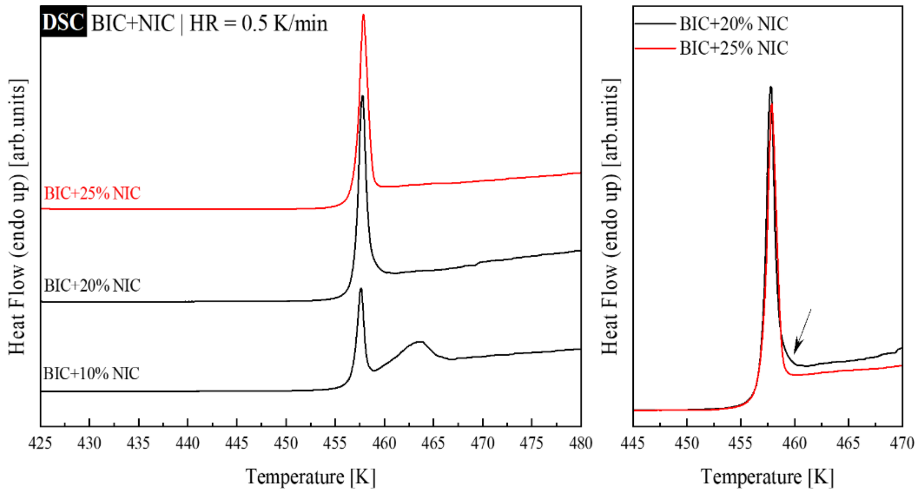

Figure S2. Differential scanning calorimetry (DSC) thermograms of co-amorphous BIC + NIC systems recorded at a low heating rate (0.5 K/min) for selected compositions. The reduced heating rate enables improved resolution of overlapping melting events, particularly in samples with low NIC content. A comparison of the eutectic composition (~25 wt.% NIC) and a neighboring composition (20 wt.% NIC) is shown on the right panel, highlighting subtle differences in melting behavior.

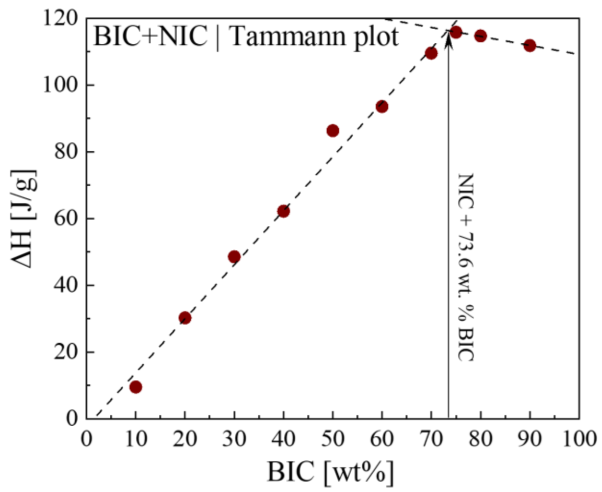

Figure S3. Tammann plot for the BIC + NIC system, presenting the enthalpy of fusion associated with the eutectic melting as a function of composition. Linear fits to the data points allow estimation of the eutectic region. The analysis confirms the location of the eutectic composition and supports the interpretation derived from DSC measurements.

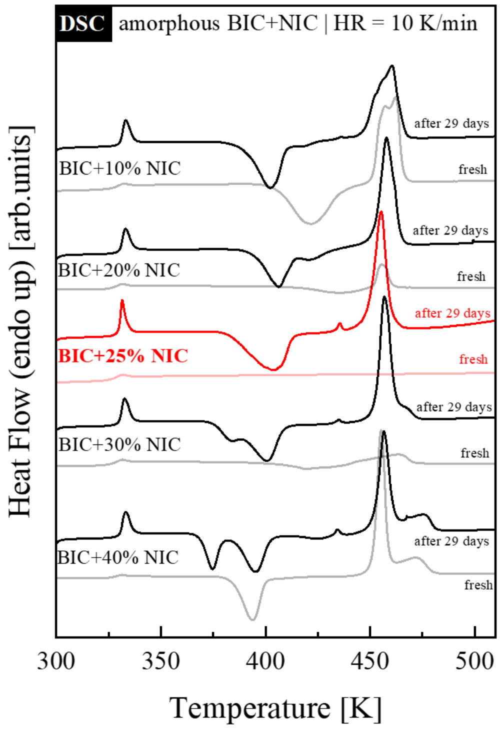

Figure S4. DSC thermograms (HR = 10 K/min) of initially amorphous BIC + NIC systems (10–40 wt.% NIC) after 29 days of storage at room temperature, compared with freshly prepared samples. All aged samples exhibit an enthalpy overshoot at T_g followed by cold crystallization and melting. Additional thermal events observed at lower temperatures may suggest the formation of additional crystalline fractions; however, due to ongoing recrystallization during heating, these features cannot be unequivocally assigned to phases formed during storage.

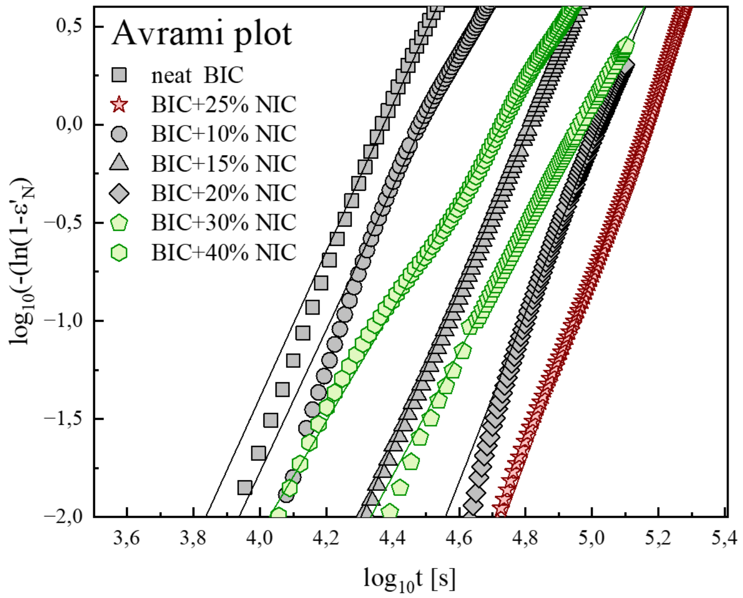

Figure S5. Linearized Avrami plots for crystallization of amorphous BIC and BIC + NIC systems (10–40 wt.% NIC), presented as $\log_{10}(-\ln(1-\varepsilon'_n))$ versus $\log_{10}(t)$. Although deviations from ideal linearity are observed, the plots allow semi-quantitative comparison of the recrystallization kinetics using the Avrami approach. The slopes of the fitted lines correspond to the Avrami exponent (n), while the horizontal shifts reflect differences in crystallization kinetics. The eutectic composition (~25 wt.% NIC) exhibits the slowest crystallization, consistent with the longest half-time values. The good linearity of the plots further supports the reliability of the extracted kinetic parameters.
